# Prevention of Wogonin on Colorectal Cancer Tumorigenesis by Regulating p53 Nuclear Translocation

**DOI:** 10.3389/fphar.2018.01356

**Published:** 2018-11-23

**Authors:** Qian Feng, Haojia Wang, Jiaying Pang, Liyan Ji, Jiada Han, Ying Wang, Xiaoxiao Qi, Zhongqiu Liu, Linlin Lu

**Affiliations:** ^1^International Institute for Translational Chinese Medicine, Guangzhou University of Chinese Medicine, Guangzhou, China; ^2^The Postdoctoral Research Station, Guangzhou University of Chinese Medicine, Guangzhou, China

**Keywords:** wogonin, colorectal cancer, p53 nuclear translocation, endoplasmic reticulum stress, apoptosis, autophagy

## Abstract

The tumor suppressor protein p53 plays an important role in the development and progression of colon cancer, and the subcellular organelle localization directly affects its function. Wogonin (5,7-dihydroxy-8-methoxyflavone), a mono-flavonoid extracted from root of *Scutellaria baicalensis Georgi*, possesses acceptable toxicity and has been used in colorectal cancer (CRC) chemoprevention in pre-clinical trials by oncologist. However, the underlying anti-colon cancer mechanisms of wogonin are not yet fully understood. In the present study, the effect of wogonin on the initiation and development of colitis-associated cancer through p53 nuclear translocation was explored. AOM-DSS CRC animal model and human CRC HCT-116 cell model were used to evaluate the *in vivo* and *in vitro* anti-colon cancer action of wogonin. We observed that wogonin showed a dramaticlly preventive effect on colon cancer. Our results showed that wogonin caused apoptotic cell death in human CRC HCT-116 cell through increased endoplasmic reticulum (ER) stress. Meanwhile, excessive ER stress facilitated the cytoplasmic localization of p53 through increasing phosphor-p53 at S315 and S376 sites, induced caspase-dependent apoptosis and inhibited autophagy. Furthermore, we verified the chemoprevention effect and toxicity of wogonin *in vivo* by utilizing an AOM-DSS colon cancer animal model. We found that wogonin not only reduced tumor multiplicity, preserved colon length to normal (6.79 ± 0.34 to 7.41 ± 0.56, *P* < 0.05) but also didn’t induce side effects on various organs. In conclusion, these results explain the anti-tumor effect of wogonin in CRC and suggest wogonin as a potential therapeutic candidate for the therapeutic strategy in CRC treatment.

## Introduction

Colorectal cancer is the third most commonly prevalent malignancy worldwide and the fourth cause of cancer-related death (Das et al., 2016). More than 1 million new cases of CRC are reported annually and the incidence rate has been increasing. In 2012, nearly 10% of the total worldwide cancers, 1,361,000 new cases were reported and nearly half died from CRC (Siegel et al., 2017). Although, the 5-year relative survival rate of CRC is on the rise, but the total death toll is still very high. Radiotherapy and chemotherapy are still preferred ways for the treatment of colon cancer. 5-fluorouracil (5-FU), leucovorin, oxaliplatin (FOLFOX) are conventional chemotherapy regimens, often fail to eradicate advanced CRC (Neska et al., 2016). Therefore, it will be a promising approach for cancer prevention and improve overall survival by utilizing food-derived, plant-derived, or pharmaceutical interventions with broad effectiveness and tolerable side effects to inhibit tumorigenesis and development.

The ER is a vital organelle and plays multifunctional roles in cellular signaling, stress response and cancer development present. Disruption of ER homeostasis is crucial for the development and maintenance of many cancers including breast (Lee et al., 2006), hepatocellular (Shuda et al., 2003), malignantgliomas (Pyrko et al., 2007), myeloma (Carrasco et al., 2007), and so on. Misfolded or unfolded proteins accumulation in the ER lumen induce ER stress, severe and prolonged ER stress has been confirmed could induce programmed cell death (Tabas and Ron, 2011; Yin et al., 2012). Apoptosis and autophagy are both forms of programmed cell death and autophagy is considered as a double-edged sword (Ouyang et al., 2012; Yu et al., 2017). It also has been reported that blockage of autophagy leads to aggravated ER stress and cell death (Burada et al., 2015; Sakitani et al., 2015).

A number of studies have shown that p53, a well-characterized tumor suppressor, gains or loses its activity depending on its localization (Iwao and Shidoji, 2014). There are several mechanisms that regulate the localization of p53 to different subcellular compartments. For instance, DNA damage and hypoxia always lead to the localization of p53 in the nucleus (Zhang et al., 2016), while ER stress and genotoxic stress could enhance cytoplasmic relocation of p53 (Qu et al., 2004). ER stress could facilitate the cytoplasmic localization of p53 through mediate directly or indirectly the phosphorylation of p53 at serine 315 or serine 376, respectively (Shi and Gu, 2012). The localization of p53 in cell could regulate the fate of cells. Through transactivation with its target genes, nuclear p53 could promote autophagy (Cheong et al., 2012). However, cytoplasmic p53 mainly inhibits autophagy through extranuclear transcription independent mechanisms (Tang et al., 2015). What’s more, Jonathan Lévy indicated that the patients who are at high risk of developing CRC could suppress cancer development and growth through the inhibition of autophagy (Levy and Romagnolo, 2016).

Wogonin, a naturally mono-flavonoid, is extracted from root of *Scutellaria baicalensis Georgi* with various activities including anti-inflammatory, anti-tumor, and anti-microbial potentials. It has been reported that wogonin could improve p53 expression in hepatoma, glioblastoma, lung and colon cancer cells (Gao et al., 2011; Kim et al., 2012; Lee et al., 2012; Qian et al., 2014). Moreover, wogonin could modulate ER stress and eventually led to autophagy and/or apoptosis. But these roles were cell-type and context dependent. For example, wogonin induced ER stress in IRE1α-dependent way in neuroblastoma, leukemia, and glioma cell lines (Tsai et al., 2012; Ge et al., 2015; Hu et al., 2015). Conversely, wogonin could reverse ER stress induced by other stressors such as wogonin inhibited tunicamycin-induced ER stress to protect dorsal root ganglion neuronsin rat (Xu et al., 2015). Therefore, it is necessary to further clarify whether the effect of wogonin on ER stress and p53 nuclear translocation to affect carcinogenesis and development of CRC.

In this study, the anti-tumor efficacy of wogonin was addressed *in vivo* by utilizing an AOM-DSS CRC animal model. The intrinsic mechanism of wogonin prevents CRC through production of ER-stress and p53 nuclear translocation was explored *in vitro* by using human CRC cell lines. Considering the important role of the intracellular localization of p53 in the development and progression of colon cancer, determining the effect of wogonin on p53 nuclear translocation may provide a new thought and method for the prevention on CRC tumorigenesis.

## Materials and Methods

### Cell Lines and Cell Culture

Human colorectal carcinoma cells HCT-116 were obtained from the American Type Culture Collection (ATCC, Manassas, VA, United States). Cell culture reagents were purchased from Invitrogen (Gibco, Waltham, MA, United States). The cells were cultured in Dulbecco’s modified eagle medium (DMEM) supplemented with 10% (v/v) fetal bovine serum (FBS), 100 U/mL penicillin and 100 μg/mL streptomycin. All cells were maintained at 37°C in a humidified atmosphere of 5% CO_2_.

### Chemicals, Reagents, and Antibodies

Wogonin (purity ≥ 98%, HPLC grade, confirmed by LC/MS/MS) was purchased from Dalian Meilun Biotech, Co., Ltd. (Dalian, China). All other chemicals were purchased from Sigma-Aldrich (St. Louis, MO, United States). Primary antibodies for LC3 I/II, Cleaved caspase 3, Cleaved caspase 9, IRE-1a, CHOP, PDI, Calnexin and secondary antibody Anti-rabbit IgG, HRP-linked were brought from Cell Signaling Technology, Inc. (Danvers, MA, United States). Primary antibody for p-p53(s15), p-p53(s376) and p-p53(s315) were purchased from Abcam, Inc. (Cambridge, MA, United States). Primary antibodies for GAPDH and Histone H3 and secondary antibody horseradish peroxidase-linked anti-mouse immunoglobulin G were purchased from Santa Cruz Biotechnology, Inc. (Santa Cruz, CA, United States). Western blot detection reagents were obtained from Bio-Rad Laboratories (Hercules, CA, United States).

### MTT Cell Viability Assay

The cell viability was measured via MTT assay. 2 × 10^3^ cells/well were seeded into 96-well plates and cultured with different doses (1∼100 μM) of wogonin for 72 h. At the end of treatment, 100 μL of MTT (0.5 mg/mL) was added to each sample and incubated for 4 h at 37°C. Then, the supernatants were discarded and 150 μL of DMSO was added to dissolve the formazan crystals. Then optical density was determined using a microplate reader Victor X3 at 490 nm (PerkinElmer, Waltham, MA, United States).

### Colony-Formation Assay

HCT-116 cells (300 cells/well) were seeded in 6-well plates 1 day before treatment. DMEM (2 ml) containing 10% FBS was added to each well with a vehicle (0.01% DMSO) or different concentration of wogonin (5 and 10 μM). After 2 weeks cultured, colonies with more than 50 cells were counted, the percentage of colony-forming rate was calculated to evaluate colony formation efficiency. Colony-formation rate was calculated as: (Colony counts experiment group/ Colony counts medium control group) × 100%.

### Apoptosis Assay

HCT-116 cells were seeded in 6-well plates (3 × 10^5^ cells/well) 1 day before treatment. Then cells were treated with or without wogonin (5 and 10 μM) for 48 h. The cells were harvested and processed for cell apoptosis analysis by using FITC Annexin V Apoptosis Detection Kit (BD Biosciences). Cell apoptosis was determined by using FACS Aria III (BD Biosciences) equipped with FlowJo v7.6. Annexin-V positive PI negative (AV^+^PI^-^) signals represent cells in early apoptosis, while Annexin-V positive PI positive (AV^+^PI^+^) signals indicate cells in late apoptosis.

### Western Blot Analysis

Cells were seeded in six-well plates with density of 4 × 10^5^ cells/well, and treated with a vehicle (0.01% DMSO) or 5 and 10 μM wogonin for 72 h. Cell lysates for protein blot analysis were prepared using standard RIPA buffer (Santa Cruz Biotechnology). Western blot analysis was performed to detect the expression levels of target proteins according to the literature (Martínez-Revelles et al., 2016). Briefly, 30 μg protein was loaded on (10%) SDS-PAGE and separated. After transmembrane, PVDF membranes were stained with primary and secondary antibodies. GAPDH or Histone H3 served as a loading control. The relative intensities of the protein bands were scanned and quantified using Quantity One Program (Bio-Rad).

### Real-Time PCR Analysis

After wogonin treatment, total RNA was isolated using TRIzol and synthesized to cDNA by using a reverse transcription kit (TaKaRa, Shiga, Japan). SYBR Green real-time PCR amplification and detection were then performed by using an ABI 7500 system (Applied Biosystems, Foster City, CA, United States). The following primers were used: DDIT3, forward 5′-AATGAACGGCTCAAGCAGGA-3′ and reverse 5′-AGCCACTTCTGGGAAAGGTG-3′; XBP1, forward 5′-AAGTTCTGCTTCTGTCGGGG-3′ and reverse 5′-TGCACGTAGTCTGAGTGCTG-3′; GAPDH, forward 5′-GGTGTGAACCATGAGAAGTATGA-3′ and reverse 5′-GAGTCCTTCCACGATACCAAAG-3′. Relative gene expression levels were normalized to GAPDH expression. The relative mRNA expression levels were analyzed by 2^-ΔΔCt^.

### Immunofluorescence Assay

The immunofluorescence assays in HCT-116 cells were seeded on 15 mm confocal dish with 5 or 10 μM wogonin treating for 48 h and fixed in cold 4% paraformaldehyde. The confocal dishes were incubated with p-p53(ser15) (Abcam, 1:200). Detection of the primary antibody was performed using 1:200 Alexa Fluor^®^ conjugated secondary antibody. The nucleus was stained with 0.1% DAPI and microfilament was stained with 0.1% phalloidin. The images were captured and analyzed using Leica TCS SP8 confocal microscope.

### Animal Model of AOM/DSS-Induced Colorectal Cancer

All animals used in this study were obtained from Laboratory Animal Center of Sun Yat-sen University (Guangzhou, China) and approved by Animal Care and Use Committee (IACUC) in Guangzhou University of Chinese Medicine. Colorectal cancer was induced in 4-week-old male C57BL/6 mice via the intraperitoneal injection of AOM (10 mg/kg) while the mice were maintained with a regular diet and drinking water for 14 days and then mice were subjected with three cycles of DSS treatment, with each cycle consisting of the administration of 2% DSS for 7 days, followed by a 14-day recovery period with regular water. Mice were administered with 50 and 100 mg/kg wogonin daily from 5^th^ to 25^th^ week.

### Statistical Analysis

All results are expressed as mean ± standard deviation (SD) of values from three independent experiments. Data were statistical analyzed by one-way ANOVA using SPSS 18.0. *P*-values < 0.05 (^∗^), < 0.01 (^∗∗^), < 0.001 (^∗∗∗^) were considered as statistical significant between experiment and control groups.

## Results

### Wogonin Induced Cell Death in Human Colorectal Cancer Cells via Caspase-Dependent Apoptotic Pathway

**FIGURE 1:**
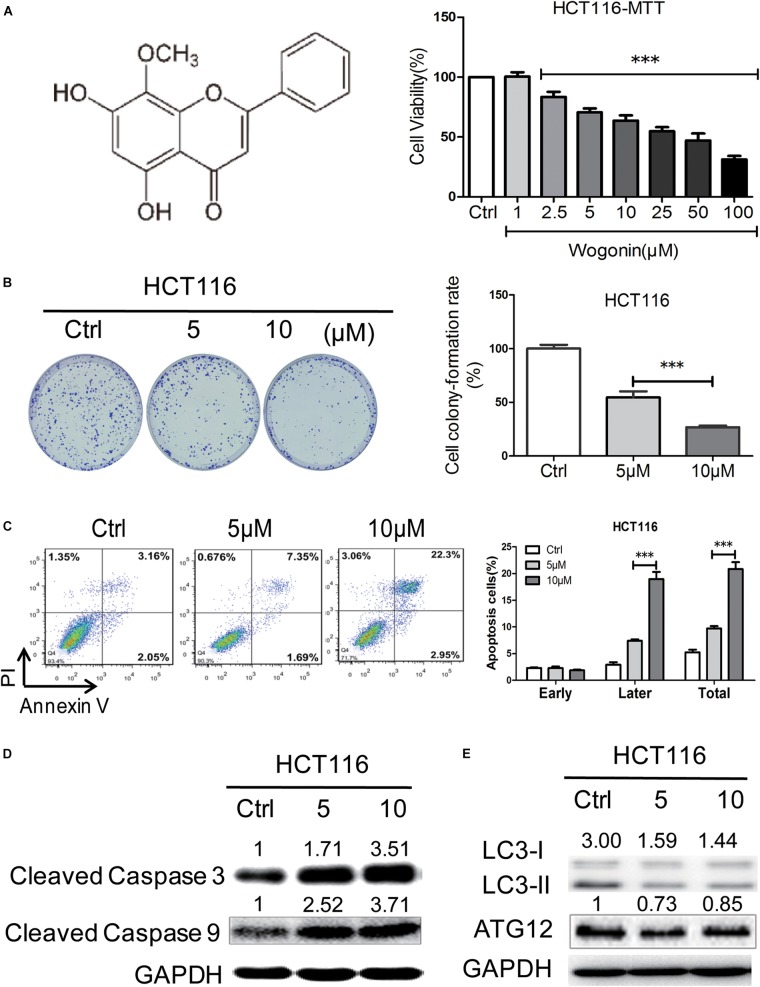
Wogonin induced cell death via caspase-dependent apoptotic pathway in human CRC cells. **(A)** Wogonin inhibited human CRC HCT-116 cells viability of in a concentration dependent manner. HCT-116 cells were treated with various concentrations (0–100 μM) of wogonin for 72 h as measured by MTT assay. **(B)** Effects of wogonin on the colony formation abilities of HCT-116 cells. Colony formation rate was showed in histogram. **(C)** Effects of wogonin on apoptotic cell death in HCT-116 cells. HCT-116 cells treated with wogonin for 48 h, and the proportion of apoptotic cells was determined by flow cytometry using Annexin V/propidium iodide (PI) double staining. **(D)** The expression levels of cleaved caspase 3 and 9 in9 in HCT-116 cells after wogonin treatment were measured using Western blot assay. GAPDH was used as an equal loading control. **(E)** Western blot analysis of autophagy-related proteins including LC3-II and LC3-I in HCT-116 cells after wogonin treatment for 72 h. GAPDH was used as an equal loading control. The results represent the mean of three independent experiments. Values represent mean ± SD, significant difference versus control group, ^∗∗∗^*P* < 0.001.

The anti-cancer effects of wogonin were first investigated *in vitro* in human colon cancer cells. Human colon cancer HCT116 cells were treated by wogonin at various concentrations (1–100 μM) for 72 h and analyzed by MTT assay. Result showed that wogonin exhibits a dose-dependent reduction in HCT116 cell viability (Figure 1A). After treatment, wogonin significantly inhibited cell growth with the maximum inhibition ratio of 68.67 ± 2.80% at 100 μM in HCT-116 cells (Figure 1A, *P* < 0.001). To further analyze the influence of wogonin in cell biology, wogonin at 5 and 10 μM, with respective cell inhibition of 29.47 ± 3.34 and 36.35 ± 4.38%, were chosen for following studies. Moreover, the anti-proliferation effects of wogonin in HCT-116 cells were evaluated by colony-formation assay. The result confirmed that wogonin inhibited HCT-116 cell productive capacity in a concentration-dependent manner (Figure 1B, *P* < 0.001), the respective cell colony formation rate is 54.58 ± 5.60 and 26.78 ± 1.55% after wogonin treatment at 5 and 10 μM for 14 days. Collectively, our results suggest that wogonin inhibits the colon cancer HCT-116 cell viability and proliferation.

To determine whether the inhibition effects of wogonin in CRC were caused by apoptosis or autophagy, flow cytometry and western blot were chosen for further analyzed. Flow cytometry analysis of AV and PI positivity showed that wogonin treatment induced significantly apoptosis in HCT-116 cells after 48 h (Figure 1C, *P* < 0.001). The percentages of late apoptotic cells were significantly increased to 7.40 ± 0.26 and 21.40 ± 0.10% after wogonin (5 and 10 μM) treatment compared with 2.93 ± 0.45% in control group. Meanwhile, in order to understand the pathway involved in wogonin-induced apoptosis, expression of cleaved caspase 3 and cleaved caspase 9, central executors of intrinsic apoptotic cell death, were detected by western blot. Protein levels of cleaved caspase 3 and cleaved caspase 9 were significantly increased by 3.52 ± 0.48 (Figure 1D, *P* < 0.01) fold and 3.72 ± 0.15 (Figure 1D, *P* < 0.001) fold respectively after wogonin (10 μM) treatment for 72 h (Figure 1D). LC3-II to LC3-I conversion, the reliable autophagy markers for monitoring autophagic cell death, also were detected by western blot. As shown in Figure 1E, we found that conversion ratio of LC3-II to LC3-I was decreased 0.49 ± 0.01 fold (*P* < 0.01) after 10 μM wogonin treatment for 72 h in HCT-116. All these data together indicated that wogonin induce human CRC HCT-116 cell death via caspase-dependent apoptotic pathway.

### Wogonin Caused Apoptotic Cell Death Through Increased Endoplasmic Reticulum Stress in Colorectal Cancer Cells

**FIGURE 2:**
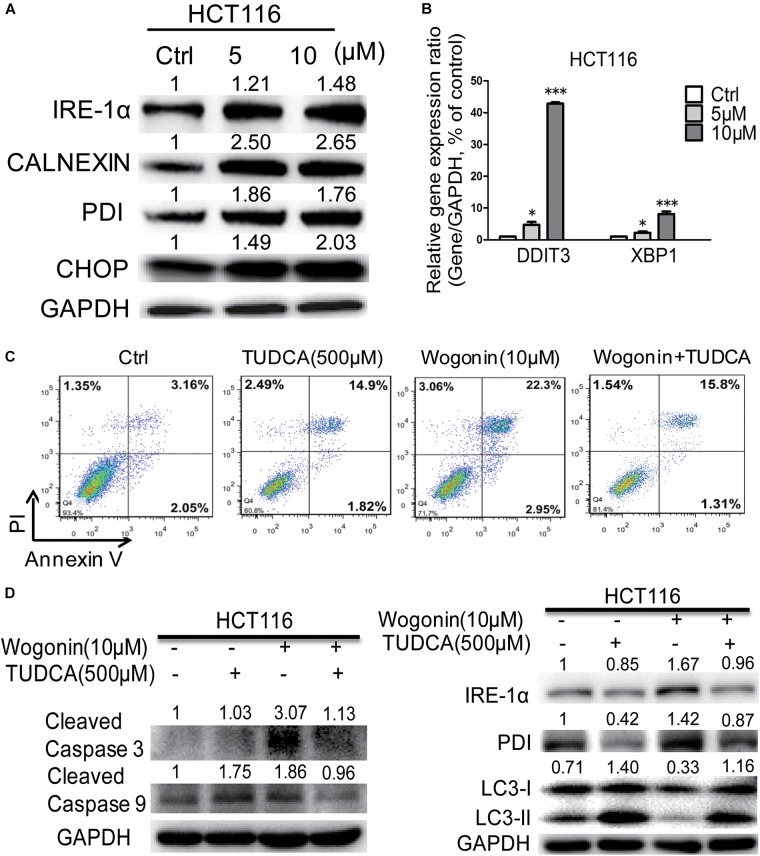
Wogonin caused apoptotic cell death through increased ER stress in human CRC HCT-116 cell. **(A)** IRE-1α, CHOP, PDI, and CALNEXIN protein levels in HCT-116 cells treated with wogonin and vesicle (0.1% DMSO) for 72 h were detected using western blot assay. The band intensities were quantified by Quantity One and represented as bar graph in the right panel. GAPDH was used as an internal control. **(B)** Gene expression of *DDIT3* and *XBP1* were determined by real-time PCR after treatments with indicated concentrations of wogonin for 72 h. *GAPDH* served as housekeeping gene. **(C)** Apoptotic effects in HCT-116 cells after wogonin treatment (10 μM) with/without TUDCA (500 μM) for 48 h, and the proportion of apoptotic cells was determined by flow cytometry using Annexin V/propidium iodide (PI) double staining. **(D)** Protein expression levels of cleaved caspase 3, cleaved caspase 9, IRE-1α, PDI, LC3-II to LC3-I in HCT-116 cells after wogonin treatment (10 μM) with/without TUDCA (500 μM) for 72 h. GAPDH was used as an equal loading control. Experiments were performed in triplicate and data represent as mean ± SD, significant difference versus control group, ^∗^*P* < 0.05; ^∗∗∗^*P* < 0.001.

It has been reported that wogonin could modulate ER stress and eventually led to autophagy and/or apoptosis (Ge et al., 2015; Hu et al., 2015). To determine whether ER stress was associated with wogonin-induced cell death, ER chaperones (CALNEXIN, PDI), ER-associated key sensors (IRE1α), downstream transcription factor CHOP and regulated genes DDIT3 and XBP1 were detected by western blotting and qRT-PCR in HCT-116 cells. As shown in Figure 2A, in HCT-116 cells, wogonin increased protein expression of PDI, CALNEXIN, IRE-1α and CHOP in a dose-dependent manner. Particularly, PDI and CALNEXIN were significantly increased by 1.76 ± 0.04 fold (*P* < 0.001) and 2.65 ± 0.11 fold (*P* < 0.001) after wogonin (10 μM) treatment. Moreover, IRE-1α showed 1.48 ± 0.03 fold increase (*P* < 0.01) after wogonin (10 μM) treatment. Furthermore, CHOP was significantly increased by 2.03 ± 0.16 fold (*P* < 0.01) after treatment with 10 μM wogonin for 72 h compared to control group. What’s more, PCR results also confirmed that mRNA levels of ER stress associated downstream gene *DDIT3* and *XBP1* in HCT-116 cells were both up-regulated by 42.91 ± 0.34 fold (Figure 2B, *P* < 0.001) and 8.10 ± 0.79 fold (Figure 2B, *P* < 0.001) after wogonin (10 μM) treatment, respectively.

Tauroursodeoxycholic acid is an ambiphilic bile acid and a specific inhibitor of ER stress (Fan et al., 2017; Fernandez-Bautista et al., 2017). Flow cytometry result showed that the apoptotic effect of wogonin (10 μM) caused in HCT-116 cells was completely reversed by TUDCA (500 μM) (Figure 2C). The percentages of apoptotic cells were significantly decreased from 25.36 ± 0.06 to 17.40 ± 0.26% after wogonin (10 μM) and TUDCA (500 μM) treatment. Meanwhile, cleaved caspase 3 and cleaved caspase 9 protein expressions were revealed 0.61 ± 0.02 fold (*P* < 0.05) and 0.52 ± 0.03 fold (*P* < 0.01) decrease after treating with TUDCA (500 μM) and wogonin (10 μM) for 72 h in HCT-116 cells (Figure 2D). IRE-1α and PDI were exhibited 0.57 ± 0.03 fold (*P* < 0.05) and 0.61 ± 0.03 fold (*P* < 0.05) decrease after treating with TUDCA (500 μM) and wogonin (10 μM). Besides that, the level of conversion ratio of LC3-II to LC3-I was increased by 3.46 ± 0.16 fold (*P* < 0.001) compare with wogonin (10 μM) treat alone for 72 h in HCT-116 cells. All these results confirmed that wogonin caused apoptotic cell death in human CRC HCT-116 cell through increased ER stress.

### Wogonin-Induced Endoplasmic Reticulum Stress Modulated Nuclear Translocation of p-p53

**FIGURE 3:**
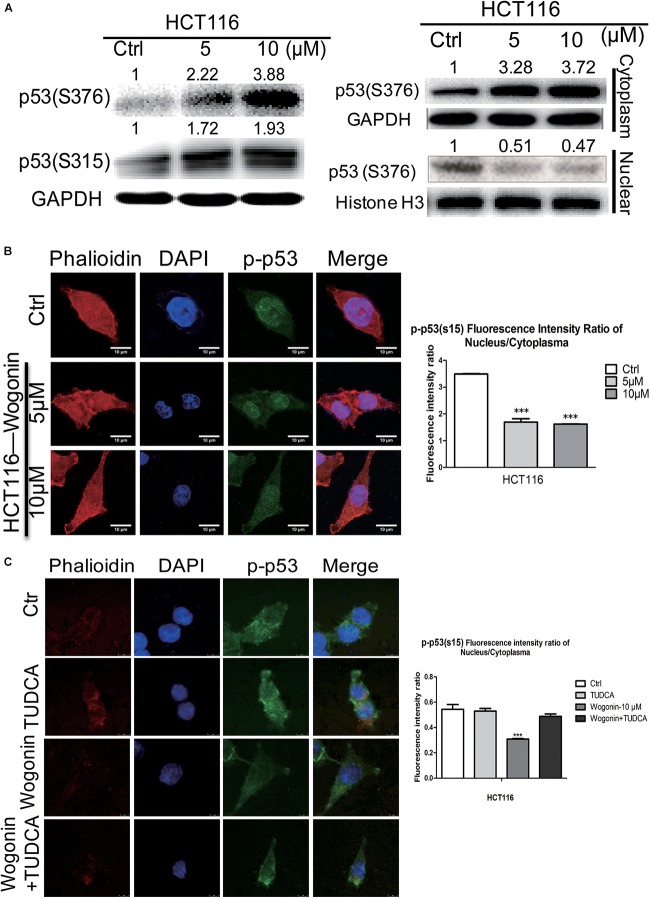
Wogonin modulated nuclear translocation of p-p53 through increased ER stress. **(A)** Total protein, cytoplasm protein and nuclear protein expressions of p53(ser315) andp53 (ser376) were examined by western blot after wogonin treated for 72 h in HCT-116 cells. Histone H3 or GAPDH was used as an internal control. **(B)** The distribution of p53(ser15) were visualized by immunofluorescence using p53(ser15) antibody (Green), nuclei were counterstained with DAPI (Blue) and cytomembrane were counterstained with phalloidine (Red). p53(ser15) fluorescence intensity ratio of cytoplasm/nucleus was expressed in histogram. **(C)** Immunofluorescence assay was conducted to evaluate the ratio of phosphorylation at serine 15 of p53 in nucleus/cytoplasm. HCT116 cells were treated with wogonin (10 μM), TUDCA (500 μM), and wogonin combined with TUDCA for 48 h in HCT-116 cells. Values represent mean ± SD, significant difference versus control group, ^∗∗∗^*P* < 0.001.

Given that ER stress was regulated by wogonin, this pathway could converge into transcription factor p53 by controlling its nucleocytoplamic shuffling (Qu et al., 2004; Shi and Gu, 2012). To investigate how p-p53 shuffle was affected by wogonin, we studied the cytoplasm/nucleus translocation of p-p53 by western blot and immunofluorescence. For total protein, after wogonin (10 μM) treatment for 72 h, phosphorylation at serine 315 and serine 376 of p53 both were increased by 1.98 ± 0.09 fold (*P* < 0.01) and 3.78 ± 0.12 fold (*P* < 0.001) in HCT-116 cells (Figure 3A). To further clear the distribution of p-p53, we separated the nuclear and cytoplasmic proteins to evaluate the localization of p-p53. In HCT-116 cells cytoplasm, wogonin treatment exhibits a dose-dependent protein expressions increase of phosphorylation at serine S376 of p53. Wogonin treatment at 10 μM exhibited 3.72 ± 0.06 fold increase (*P* < 0.001) compare to control group. While in nucleus, expressions of S376 of p53 were decreased to 0.51 ± 0.02 (*P* < 0.05) fold and 0.47 ± 0.04 fold (*P* < 0.01) after wogonin (5 and 10 μM) treatment (Figure 3A).

Immunofluorescence assay was used to further confirm whether wogonin could influence the cytoplasmic/nuclear distribution of phosphorylation of p53. In HCT-116 cells, phosphorylation at serine 15 of p53 in nucleus was dramatically decreased, and the ratio of phosphorylation at serine 15 of p53 in nucleus/cytoplasm was decreased to 0.47 ± 0.002 fold (*P* < 0.001) after wogonin treatment for 48 h (Figure 3B). At the same time, ER stress inhibitor, TUDCA (500 μM) completely reversed the decreased effect of the ratio of phosphorylationof p53 in nucleus/cytoplasm caused by wogonin (10 μM) (Figure 3C). These findings suggested that wogonin regulated nuclear translocation of p-p53 by increasing the ER stress response to inhibit autophagy in HCT116 colon cancer cells.

### Wogonin Inhibited Tumorigenesis in AOM/DSS-Induced CRC Mice Model Through Increasing Endoplasmic Reticulum Stress

**FIGURE 4:**
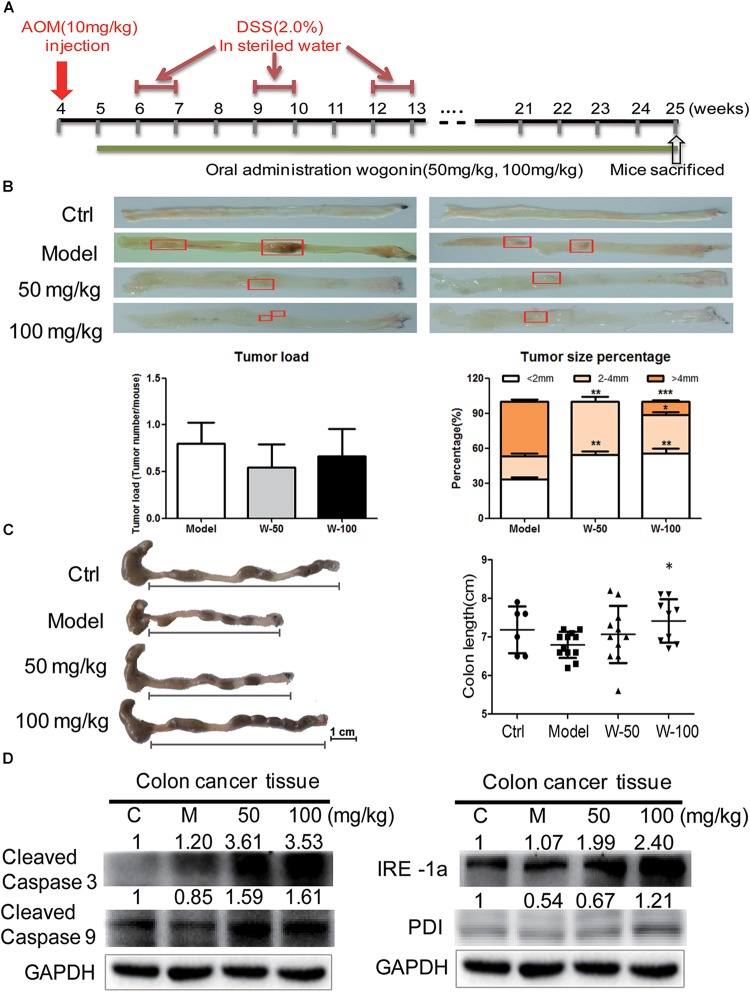
Wogonin inhibited tumor carcinogenesis in AOM/DSS induced CRC. **(A)** Schematic diagram of AOM/DSS-induced colitis associated colon cancer model (*n* = 15 for each group). **(B)** The cutaway view of mice colon tissue and the number of tumor load at the endpoint of experiment, tumor size of colonic neoplasms in each group. **(C)** The colon length changes were measured by using a scaled ruler in each group. **(D)** Apoptotic and ER-stress related proteins expression after wogonin treatment (50 and 100 mg/kg) for 21 weeks in mice were determined by western blot. GAPDH was used as an equal loading control, significant difference versus control group, ^∗^*P* < 0.05; ^∗∗^*P* < 0.01; ^∗∗∗^*P* < 0.001.

The AOM-DSS colon cancer animal model was used to evaluate the chemopreventive effect and toxicity of wogonin *in vivo.* As shown in Figure 4A, mice developed tumors when they were given with AOM (10 mg/kg) by i.p. injection at week 4 and fed with DSS (2%) water for three-cycles. After consecutive 21 weeks of wogonin oral administration, there was no significant body weight loss in mice (Supplementary Figure 1A). At the termination of experiment, tumor node multiplicity was calculated as tumors stratifying by size according to diameter, tumor incidence is reduced from 80.00% in model group to 54.55 and 66.67% in 50 and 100 mg/kg wogonin treatment group, tumor multiplicity with size of <2 and >4 mm were 55.55 and 11.11% in mice treated with 100 mg/kg wogonin compared with model group 33.34 and 46.67%, respectively (Figure 4B). Concurrently, wogonin also preserved colon length to normal from 6.79 ± 0.34 cm in model group to 7.06 ± 0.74 and 7.41 ± 0.56 (*P* < 0.05) cm in wogonin (50 and 100 mg/kg) treated groups (Figure 4C).

To determine the effect of wogonin *in vivo*, western blot was performed to detect apoptotic markers in wogonin treatment groups as compared with model group. Protein expression of cleaved caspase 3 and cleaved caspase 9 were significantly increased by 3.53 ± 0.13 (*P* < 0.001) and 1.61 ± 0.16 (*P* < 0.05) fold respectively in 100 mg/kg wogonin treated group. To verify the potential mechanism of wogonin *in vivo*, ER stress markers were measured in colon tissue of mice with AOM/DSS induced colon cancer. Results showed that, protein level of PDI was increased by 2.34 ± 0.19 (*P* < 0.001) fold, and IRE1α was significantly increased by 2.24 ± 0.14 (*P* < 0.001) fold in intestine of mice treated with 100 mg/kg wogonin as compare with model group (Figure 4D).

## Discussion

Colitis-associated CRC is a chronic inflammation disorder eventually developed into cancer in bowel. Colitis-associated colon cancer is a preventable malignancy, and phytochemicals such as flavonoids (wogonin, curcumin, and quercetin) have been used in CRC chemoprevention in pre-clinical trials by oncologist. Although wogonin has been extensively studied as an anti-tumor flavonoid with acceptable toxicity, not much is known about whether wogonin could prevent colitis associated CRC. In this study, the chemoprevention and chemotherapeutic effects of wogonin were determined and the p53 nucleocytoplamic shuffling involved mechanism was subsequently evaluated. We found that wogonin induces human CRC HCT-116 cell caspase-dependent apoptosis through increased ER stress. Subsequently, excessive ER stress inhibited translocation of p53 into the nucleus through increasing phosphor-p53 at S315 and S376 sites, which eventually suppressed and prevented the development of CRC.

Herein, we used AOM-DSS induced CRC mouse model, resemble to human colitis-associated CRC, and found that wogonin exhibited chemopreventive effect *in vivo*. We found that wogonin successfully prevents CRC tumorigenesis through significantly reducing tumor multiplicity with size of > 4 mm from 46.67 to 11.11% (Figure 4B, *P* < 0.001). Meanwhile, wogonin reversed colon length to normal from 6.79 ± 0.34 cm in AOM/DSS group to 7.41 ± 0.56 cm in 100 mg/kg wogonin treatment group (Figure 4C, *P* < 0.05). The most important is that wogonin did not show obvious toxicity and there is no significant change in the organ index between mice administrated with wogonin and those with saline (Supplementary Figure 1). These findings indicate wogonin exerts chemopreventive effect without systemic toxicity in colitis-associated CRC.

Next, we explored the underlying mechanism of wogonin exerts chemopreventive effects *in vitro*. We found that wogonin significantly inhibits the viability and proliferation (Figure 1, *P* < 0.001) in HCT-116 colon cancer cells. Moreover, wogonin specifically induced human CRC HCT-116 cell apoptosis via caspase-dependent pathway (Figure 1D, *P* < 0.05). What’s more, wogonin caused apoptotic cell death through dose-dependently activated ER stress pathway both *in vivo* and *in vitro* by increasing ER chaperones (CALNEXIN, PDI), ER-associated key sensors (IRE1α), downstream transcription factor CHOP protein expressions (Figures 2A, 4, *P* < 0.05) and targeted genes *DDI3* and *XBP1* mRNA expressions (Figure 2B, *P* < 0.001). Previous study revealed that ER stress could directly or indirectly mediate the phosphorylation of p53 and the phosphorylationof p53 at nuclear localization sequence (305–321 bp) sequence blocked the nuclear translocation (Shi and Gu, 2012). In good agreement with previous study, our results further demonstrated that excessive ER stress induced by wogonin ultimately facilitated the cytoplasmic localization of p53 through increasing phosphor-p53 at S315 and S376 sites (Figures 3A,C, *P* < 0.01). Meanwhile, wogonin concentration-dependently reduced the ratio of phosphorylation at serine 15 of p53 in nucleus/cytoplasm (Figure 3B, *P* < 0.001). Previous study demonstrated that cytoplasmic p53 could inhibit autophagy (Cheong et al., 2012), our study also confirmed that wogonin treatment exhibited the effect of inhibiting autophagy (Figures 1E, 2D, *P* < 0.05). Potentially, wogonin could have stabilized p53 in the cytoplasm by blockings its export and/or degradation thereby leading to increased levels of total p53 and the localized cytoplasm of p53 induced caspase-dependent apoptosis and inhibited autophagy.

In summary, we found that wogonin could prevent colitis-associated colon cancer in AOM/DSS induced mice model. Multiple pathways were involved in chemopreventive effect of wogonin. These findings provided wogonin as a promising agent for colitis-associated colon cancer chemopreventive agent.

## Conclusion

Taken together, by determining regulation p53 nuclear translocation mechanism of wogonin in CRC, our present study advanced our understandings of ER stress directly mediate the phosphorylation of p53 and the anti-tumor effect of wogonin in following aspects: (i) Subcellular localization of p53 regulates the activity of p53 and affects its function to modulate cellular growth, which implied that regulation of p53 nucleocytoplasmic shuttling offers a novel avenue for the therapeutic strategy in cancer; (ii) Wogonin suppressed CRC tumorigenesis suggested that wogonin is a promising therapeutic candidate for the therapeutic strategy in CRC treatment; (iii) Our mechanistic study uncovered the vital role of wogonin predominantly increased ER stress, which directly mediated the phosphorylation of p53 and ultimately facilitated the cytoplasmic localization of p53, while simultaneously accelerate cellular apoptosis and inhibit autophagy program. The results we confirmed laid a solid pre-clinical foundation for the therapeutic strategy of wogonin in the treatment of CRC through regulating p53 nucleocytoplasmic shuttling.

## Author Contributions

LL and ZL conceived the experiments and supervised all research. HW, QF, and JP performed the experiments, analyzed the data, and prepared the draft of the manuscript. LL and QF wrote and revised the manuscript. LJ, JH, YW, and XQ provided some technical support. All the authors are accountable for the content of the work.

## Conflict of Interest Statement

The authors declare that the research was conducted in the absence of any commercial or financial relationships that could be construed as a potential conflict of interest.

## Abbreviations

AOM: azoxymethane
CHOP: C/EBP homologous protein
CRC: colorectal cancer
DSS: dextran sodium sulfate
ER: endoplasmic reticulum
IRE1α: inositol requiring enzyme 1α
PDI: protein disulfide isomerase
TUDCA: tauroursodeoxycholic acid
