# Supplementary material for: Prevention of Wogonin on Colorectal Cancer Tumorigenesis by Regulating p53 Nuclear Translocation
Source: Front Pharmacol. 2018 Nov 23;9:1356. doi: 10.3389/fphar.2018.01356 (PMC6265339; doi:10.3389/fphar.2018.01356)
Supplement: Supplementary file 1 [file Presentation_1.PPTX]

## Slide 1
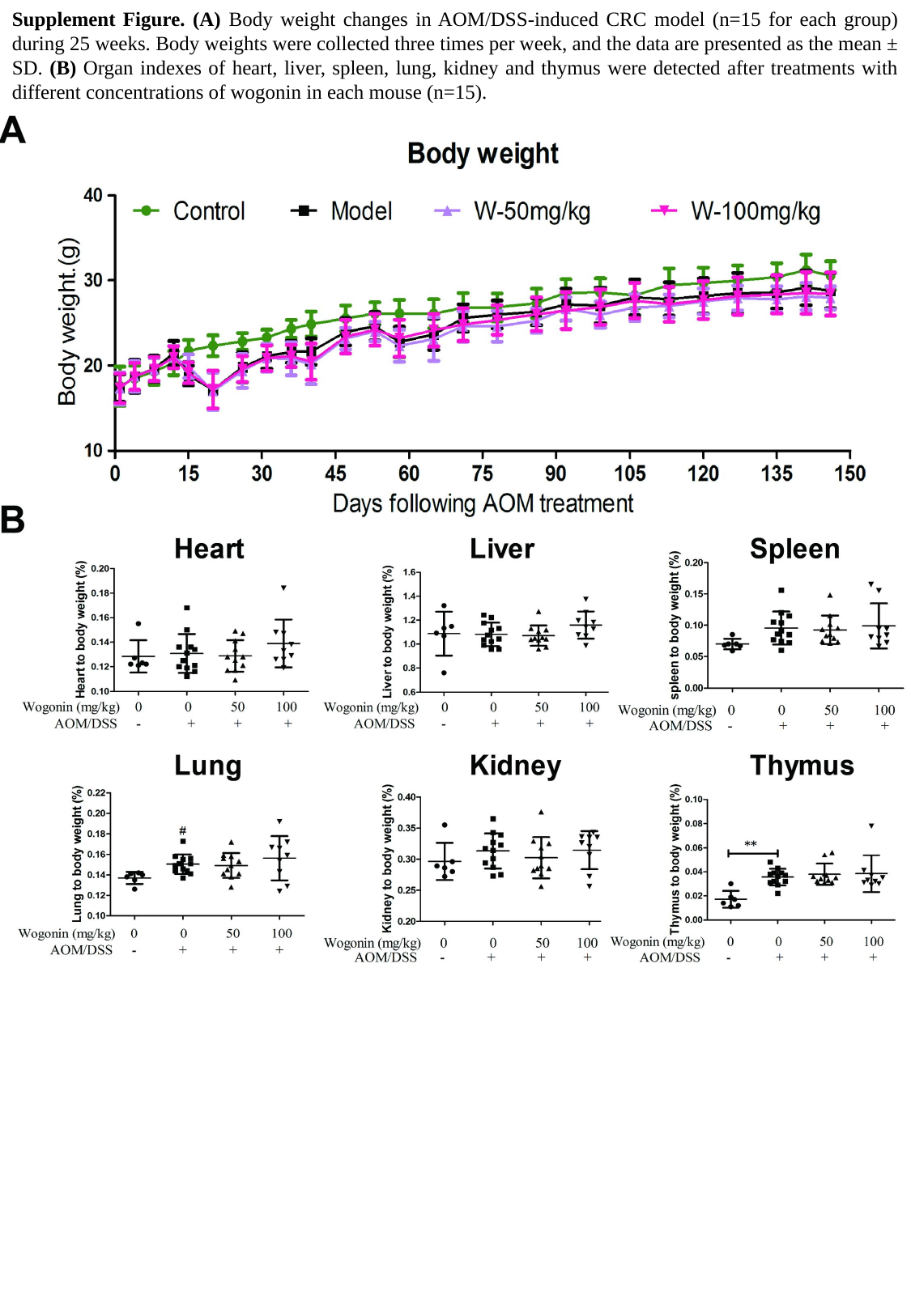

Supplement Figure. (A) Body weight changes in AOM/DSS-induced CRC model (n=15 for each group) during 25 weeks. Body weights were collected three times per week, and the data are presented as the mean ± SD. (B) Organ indexes of heart, liver, spleen, lung, kidney and thymus were detected after treatments with different concentrations of wogonin in each mouse (n=15).
